# The Role of Macrophages in Acute Pancreatitis: From Inflammatory Drivers to Therapeutic Targets

**DOI:** 10.7150/ijms.111818

**Published:** 2026-05-01

**Authors:** Zhihong Xu, Yi-Chen Sui, Li-Ping Sheng

**Affiliations:** 1 Department of Emergency, the First Affiliated Hospital of Xiamen University, School of Medicine, Xiamen University, Xiamen, China.; 2 College of Medicine, Tarim University, Alar, Xinjiang, China.; 3 Department of Gastroenterology, the First Affiliated Hospital of Xiamen University, School of Medicine, Xiamen University, Xiamen, China.

**Keywords:** acute pancreatitis, macrophage, macrophage polarization, apoptosis, pyroptosis, ferroptosis, autophagy

## Abstract

Acute pancreatitis (AP) involves activation of the innate immune system and an imbalanced inflammatory response. Both pancreatic and systemic inflammation associated with AP are mediated by macrophages. This review summarizes research on macrophage functions in AP pathogenesis and treatment. Relevant studies investigating macrophage activation, phenotypes, autophagy, and their roles in local and systemic inflammation were analyzed. During AP, macrophages initially adopt a pro-inflammatory phenotype to amplify inflammation. Macrophages located in tissues including the pancreas, adipose tissue, liver, lung, and spleen play a role in this process. As the disease progresses, they transition to an M2 phenotype to promote resolution. Macrophage-centered modulation of the innate immune response represents a promising therapeutic strategy. However, further exploration of macrophage interactions with other immune cells and pancreatic acinar cells is still needed. Targeting specific macrophage functions may improve AP management, which is the focus of this review.

## 1. Introduction

Acute pancreatitis (AP) is among the primary reasons for hospitalization related to gastrointestinal diseases, with severity and duration varying widely 1. AP can trigger an inflammatory cascade that affects multiple organs, particularly in severe acute pancreatitis (SAP) 2. The primary causes of AP include alcohol consumption, hypertriglyceridemia (HTG), pancreatic or biliary duct blockages, and other causes 3. These triggers initiate pathological cellular pathways and organelle malfunctions, leading to pancreatic acinar cell death and pancreatic inflammation 4, 5. Abnormally elevated Ca^2+^ levels in pancreatic acinar cells, caused by the non-oxidative products of alcohol, fatty acids, bile acids, and ductal obstruction, lead to pancreatic acinar cell necrosis in AP 6-8. Moreover, the activation of premature trypsinogen can also contribute to pancreatic acinar cell necrosis 9, 10. What are the mechanisms through which pancreatic acinar necrosis induces both local and systemic inflammation?

Pancreatic acinar cells drive pancreatic and systemic inflammation by activating various inflammatory cells. Previous reviews on pancreatitis have focused on the roles of multiple immune cells, highlighting their collaborative actions as well as the inflammatory processes mediated by macrophages, particularly the M1/M2 polarization 11-13. This review highlights recent advances in macrophage biology in AP and, for the first time, examines the evidence linking specific molecular signaling pathways to macrophage functional states and clinical outcomes, with an emphasis on translational implications.

## 2. The role of macrophages in connecting pancreatic inflammation to systemic inflammation in AP

Macrophages exist in the pancreas since the embryonic stage 14. During pancreatitis, tissue-resident macrophages in the pancreas show a notable increase in number 15. These resident macrophages include islet macrophages, which play roles in metabolic disorders. In obesity, for example, islet macrophages disrupt the insulin-secretion function of β-cells 16. In addition to resident macrophages, the pancreas also contains recruited macrophages. Damaged pancreatic acinar cells release chemokines that attract monocytes to the pancreas 4.

Macrophages are equipped with diverse receptors that allow them to detect various environmental cues, including hypoxia, pressure, and metabolites such as fatty acids 17. Macrophage activation is triggered by damage-associated molecular patterns (DAMPs) released from necrotic or injured pancreatic acinar cells. This activation triggers the release of pro-inflammatory cytokines, which recruit more immune cells to the injury site 18.

Activated macrophages play a pivotal role in both pancreatic and systemic inflammation during AP, contributing to the tissue damage in the pancreas and other organs. The activation of macrophages in distant organs exacerbates systemic inflammation and contributes to organ dysfunction. However, the specific mechanisms underlying this distal injury remain poorly understood 19.

## 3. Activators of macrophages in AP

The immune system, involving macrophages, is activated during AP, leading to the release of inflammatory factors that promote disease progression. The activation of macrophages in AP is driven by various substances released from pancreatic acinar cells, including DNA, ATP, chemokine CXCL10, long non-coding RNA (lncRNA), and microRNA (miRNA). Pancreatic acinar cell damage is central to the pancreatic injury observed in AP. In mouse models of AP, DNA released from pancreatic acinar cell death activates the macrophage cGAS-STING signaling pathway, triggering IRF3 and NF-κB activation, which upregulate the expression of inflammatory genes such as IFNβ and TNF-α 20. Additionally, experimental AP studies in animals indicate that pancreatic macrophages detect DNA fragments from necrotic or injured pancreatic acinar cells via TLR9 receptors, which initiate NF-κB translocation and pro-IL-1β transcription. Moreover, ATP released from pancreatic acinar cells activates the NLRP3 inflammasome through the P2X7 receptor, converting pro-IL-1β into active IL-1β 18. CXCL10, released from pancreatic acinar cells, drives M1 polarization of pancreatic macrophages in AP, promoting pro-inflammatory responses 21. In mouse models of AP, pancreatic acinar cell-derived extracellular vesicles (EVs) containing molecules such as lncRNA MALAT1 have also been implicated in promoting M1 macrophage polarization through the miR-181a-5p/HMGB1 regulatory axis 22. Furthermore, EVs derived from pancreatic acinar cells containing miR-183-5p exacerbates AP by driving M1 macrophage polarization via the reduction of FoxO1 expression 23. Additionally, miR-125b-5p from AR42J acinar cell-derived exosomes exacerbates AP by hindering M2 macrophage polarization through the PI3K/AKT pathway 24. The miR-24-3p contained in exosomes derived from pancreatic acinar cells, stimulated by cerulein, also induces M1 polarization and pyroptosis of peritoneal macrophages through the MARCH3/NLRP3 pathway in AP 25.

In addition to macrophage activation by pancreatic acinar cell damage or necrosis in AP, other factors such as trypsinogen, lipids, and hyperglycemia can also stimulate macrophages. Trypsinogen activation within macrophages during pancreatitis triggers macrophage activation through NF-κB-translocation 26. In obese mice, free fatty acids produced by adipocyte lipolysis exacerbate SAP by activating the NLRP3-caspase1 inflammasome pathway in adipose tissue macrophages (ATMs) 27. Lipids from necrotic adipose tissue interfere with M2 polarization and promote the M1 phenotype in macrophages, further amplifying the inflammatory response in AP 28. Using an AP mouse model, researchers found that hyperglycemia exacerbates AP by inducing M1 polarization of pancreatic macrophages through enhanced Notch signaling 29.

## 4. The varied roles of macrophages in pancreatic and extra-pancreatic tissues during AP

Macrophages, as key players in the immune system, play crucial roles in different tissues during AP. HTG increases macrophage infiltration in mouse pancreatic tissue 30. Additionally, ATMs gather in obesity and promote the production of pro-inflammatory cytokines 27, 31. During obesity, the increased accumulation of ATMs in adipose tissue mainly arises from CCR2-dependent recruitment of circulating monocytes, and can also result from local expansion of tissue-resident macrophages 32, 33. Both HTG and obesity significantly increase the risk of poor prognosis in AP, exacerbating AP inflammation through macrophages 27, 30, 34, 35. In obesity-related SAP, plasma exosomes promote M1 polarization in ATMs, worsening the severity of pancreatitis 36.

Macrophages in extra-pancreatic tissues, such as the liver, peritoneum, intestine, lung, and spleen, also play significantly roles in modulating AP progression and treatment response. Their functional diversity, especially their polarization states, significantly influences the pathogenesis, progression, and complications of AP. For example, liver macrophages in SAP rats undergo a polarization shift from M1 to M2 in vitro, suggesting potential therapeutic avenues for modulating the shift 37. Abdominal paracentesis drainage (APD) improves SAP in rats by promoting M2 polarization in peritoneal macrophages, highlighting an important therapeutic approach 38. In a rat SAP model, APD also enhances M2 polarization in intestinal macrophages by inhibiting the ASK1/JNK pathway, further contributing to its therapeutic benefits for intestinal damage associated with SAP 39. Furthermore, modulating macrophage polarization in the injured lung during SAP may serve as a potential therapeutic target 19, 40. Tuftsin, a natural tetrapeptide (Thr-Lys-Pro-Arg) secreted by the spleen, enhances anti-inflammatory M2 macrophage polarization by upregulating NRP1 on macrophages in the spleen during SAP 41. These studies collectively emphasize the critical and varied roles of macrophages in different tissues during SAP, offering valuable insights into potential treatment strategies.

## 5. Mechanisms of macrophage M1 polarization in AP

As previously indicated, macrophage polarization significantly influences the pathophysiology of AP 42. In mouse AP models, macrophages regulate AP inflammation through their functional plasticity, exhibiting dynamic shifts between M1 and M2 phenotypes 43. Pro-inflammatory M1 macrophages are key drivers of AP inflammation, and reducing macrophage infiltration in the pancreas can alleviate disease severity 44.

Although various molecules and vesicles, such as MLKL, HMGB1, FoxO1, lipids, Notch, STING, serum exosomes, NLRP3, CARD9-NF-κB, and STAT3, are involved in regulating M1 polarization, the hierarchical organization, crosstalk, and dynamic interplay among these factors remain poorly understood. This section reviews the current mechanisms of M1 polarization in AP, highlighting key regulatory axes. For example, the suppression of the MLKL-CXCL10 axis attenuates AP severity in mice by reducing M1 polarization of pancreatic macrophages 21. Furthermore, MALAT1, carried by EVs, enhances M1 polarization by regulating the miR-181a-5p/HMGB1 signaling axis 22. Additionally, EV-derived miR-183-5p suppresses FoxO1 expression, driving M1 macrophage polarization that exacerbates AP 23. In AP, lipids released from necrotic adipose tissue can promote the M1 phenotype in macrophages, though the underlying mechanisms remain unclear 28. Hyperglycemia exacerbates AP by promoting pancreatic macrophage M1 polarization via enhanced Notch signaling after AP 29. Inhibiting the STING pathway ameliorates lung injury associated with SAP by reversing macrophage M1 polarization 19. Obesity exacerbates SAP severity by promoting M1 polarization in ATMs through serum exosomes, thereby amplifying the immune response in adipose tissue 36. Activation of the NLRP3 inflammasome pathway in macrophages also promotes M1 polarization 45. Moreover, mesenchymal stem cells (MSCs) influence peritoneal macrophage M1 polarization during SAP through the CARD9-NF-κB pathway 46. In mouse models of AP, STAT3 phosphorylation worsens pancreatic damage by inducing M1 polarization of macrophages 47.

These molecules and pathways can be regarded as different layers and interaction nodes that regulate macrophage M1 polarization. Upstream triggers often arise from DAMPs. HMGB1, as a prototypical DAMP, can activate Toll-like receptors and NF-κB signaling to promote inflammation 48, while STING senses intracellular DNA and initiates pro-inflammatory gene expression, often acting as an upstream driver of inflammation 49. MLKL-mediated necroptosis can release additional DAMPs, thereby amplifying STING-related pathway activation 50. Downstream effectors include inflammasomes and transcriptional modules: the NLRP3 inflammasome activates caspase-1, and promotes secretion of pro-inflammatory cytokines IL-1β 51; the CARD9-NF-κB axis and STAT3 serve as core transcriptional regulators that directly upregulate M1-associated genes 46, 47, 52, 53. Notch signaling can act as a regulator of M1 polarization and also engage in crosstalk with NF-κB to modulate polarization 29, 54. Thus, these pathways do not operate in isolation: HMGB1/STING/MLKL-type upstream events enhance inflammatory inputs that activate NLRP3 and CARD9-NF-κB, which, together with Notch signaling, cooperate to drive M1 polarization.

## 6. Mechanisms of transition to M2 macrophages in resolving inflammation

As AP progresses, a shift toward the M2 phenotype occurs, characterized by the expression of anti-inflammatory cytokines such as IL-4 and IL-10. The transition plays a vital role in inflammation resolution and tissue repair 38, 55. M2 macrophage polarization involves multiple molecules and signaling pathways, such as miRNAs, PI3K/AKT, ASK1/JNK, IRF5, TSG-6, and ERK/MAPK. Exosomal miR-125b-5p from AR42J acinar cells exacerbates AP by suppressing M2 macrophage polarization through the PI3K/AKT pathway 24. APD effectively reduces the severity of inflammation in SAP by promoting M2 polarization in peritoneal macrophages 38. APD also facilitates the transition of intestinal macrophages to the M2 phenotype by suppressing the ASK1/JNK pathway, thereby reducing damage to the intestinal mucosal barrier 39. Conversely, molecules like IRF5 have been shown to suppress the M2 phenotype in lung macrophages during AP 40. MSCs promote M2 polarization by secreting TSG-6 56. In addition, in mouse models of SAP, the highly active isoflavone tectoridin promotes M2 polarization in macrophages by inhibiting the ERK/MAPK signaling pathway, thereby reducing pancreatic injury 57.

Despite the diversity of upstream triggers and signaling molecules governing macrophage polarization, many pathways converge on inflammatory transcriptional programs or on metabolic and autophagic regulators, suggesting that metabolic reprogramming and transcriptional activation together determine the M1 or M2 phenotype. Macrophage polarization plays a pivotal role in AP, as it significantly impacts the inflammatory response. Elucidating the functions of macrophages and their regulatory mechanisms in AP could help develop new therapeutic strategies to reduce inflammation and promote tissue repair. However, most study on these mechanisms are based on mouse or rat models, which may not fully replicate the immune microenvironment in human AP. Future work should concentrate on further elucidating the detailed molecular mechanisms underlying macrophage polarization, as well as how these cells interact with other immune cells and pancreatic acinar cells. Additionally, more emphasis should be placed on validating signaling pathways using human specimens. This comprehensive approach will advance our knowledge of the complex pathophysiology underlying AP.

## 7. The role of other macrophage phenotypes and autophagy in AP

In addition to macrophage polarization, cell death phenotypes (such as apoptosis, pyroptosis, and ferroptosis) along with autophagy, are involved in the inflammatory damage observed in AP. Apoptosis plays a role in the development of acute lung injury (ALI) associated with SAP in rat models 58. Inhibiting STING reduces SAP-associated ALI by preventing macrophage apoptosis 19. In AP mice, CD36-positive macrophages protect by clearing apoptotic pancreatic acinar cells 59. However, gadolinium chloride treatment significantly improves SAP-ALI by enhancing the apoptosis rate of alveolar macrophages 60.

The NLRP3 inflammasome regulates macrophage pyroptosis. Numerous studies have demonstrated that NLRP3 inflammasome-dependent pyroptosis plays a key role in the development of AP 61. In AP, exosomes containing miR-24-3p, released from caerulein-stimulated pancreatic acinar cells, induce pyroptosis in peritoneal macrophages through the MARCH3/NLRP3 pathway 25. Activation of the NLRP3 inflammasome in alveolar macrophages, leading to pyroptosis, contributes to lung damage following AP 62. Moreover, inhibiting any component of the mtDNA-cGAS-STING-IRF7/IRF3 axis suppresses the activation of NLRP3 inflammasomes, reducing the pyroptosis of alveolar macrophages and ameliorating SAP-associated ALI in mice 63. Additionally, emodin alleviates AP-induced ALI by suppressing the pyroptosis of alveolar macrophages 64.

Ferroptosis, a form of iron-dependent cell death caused by phospholipid peroxidation, is closely associated with lipid peroxidation and reactive oxygen species, both of which play pivotal roles in the pathogenesis of AP. The pancreas functions not only as a digestive organ but also as a primary site for iron storage, which is closely linked to ferroptosis 65. Ferroptosis has been shown to exacerbate the severity of pancreatitis in mice 66. However, research on the role of macrophage ferroptosis in AP is still limited.

Autophagy also plays a crucial role in modulating the inflammatory response. In experimental pancreatitis, autophagy is activated in pancreatic acinar cells, but its completion is inhibited, suggesting impaired autophagic flux. This dysfunction in autophagy enhances macrophage infiltration into the pancreas 67, 68. Additionally, in SAP mouse models, macrophage-derived p38α has been shown to worsen disease severity by modulating autophagy 69. Disrupted autophagic flux in ATMs is likely to intensify pancreatic damage in obesity-related SAP 70, 71.

## 8. Conclusion and prospects

**Figure 1:**
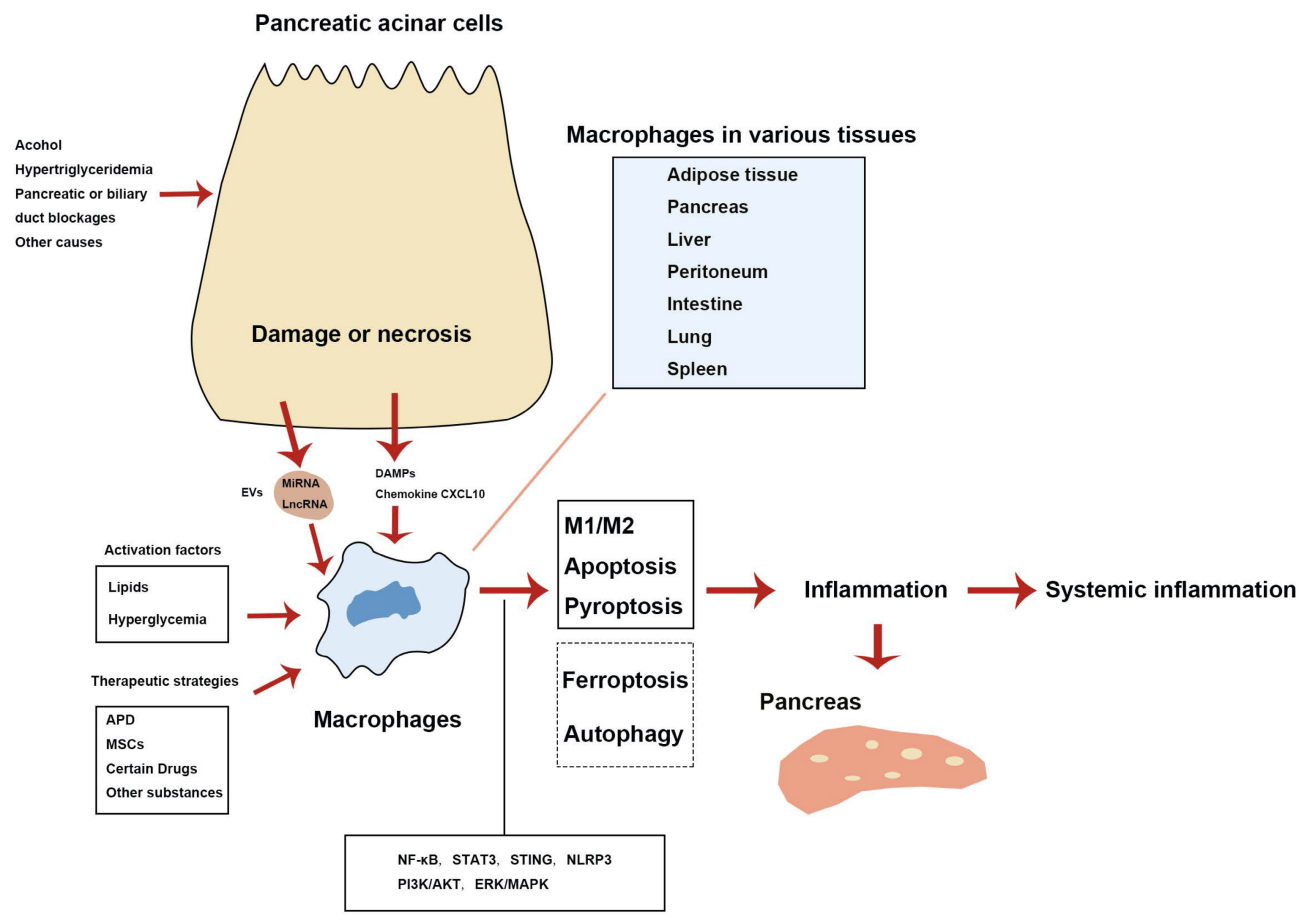
** Mechanisms of various macrophage phenotypes and autophagy involved in the inflammatory response during AP.** Solid lines denote features found in AP according to published research. Dashed lines indicate that macrophage ferroptosis and autophagy regulation remain incompletely confirmed. APD, abdominal paracentesis drainage; DAMPs, damage-associated molecular patterns; EVs, extracellular vesicles; lncRNA, long non-coding RNA; miRNA, microRNA; MSCs, mesenchymal stem cells.

In summary, macrophages are central to the pathogenesis of AP by driving both pancreatic and systemic inflammation. Damaged pancreatic acinar cells activate macrophages through various DAMPs and EVs, which push macrophages towards a pro-inflammatory phenotype. In addition, factors such as lipids, including free fatty acids, and hyperglycemia can also stimulate macrophage activation. These activated macrophages amplify the inflammatory response and tissue damage by releasing pro-inflammatory mediators. Therapeutic strategies, including APD, MSCs, and specific drugs, promote the polarization of macrophages towards an anti-inflammatory M2 phenotype, which is crucial for inflammation resolution and tissue repair. Modulating macrophage polarization offers promising therapeutic opportunities in managing AP. Besides macrophage polarization, cell death phenotypes (including apoptosis, pyroptosis, and ferroptosis) and autophagy contribute to the inflammatory damage observed in AP. Figure 1 summarizes the key mechanisms of various macrophage phenotypes and autophagy in the AP inflammatory response.

Despite significant progress, further research is essential to comprehensively understand the complex mechanisms underlying macrophage phenotypes, such as polarization, apoptosis, pyroptosis, and ferroptosis, as well as the role of autophagy across various tissues during AP, especially regarding ferroptosis and autophagy. It is also important to explore the interactions among macrophages, pancreatic acinar cells, and other immune cells. To facilitate clinical translation, future work should validate key signaling pathways in human specimens rather than relying solely on rodent models.

## Abbreviations

ALI: acute lung injury
AP: acute pancreatitis
APD: abdominal paracentesis drainage
ATMs: adipose tissue macrophages
DAMPs: damage-associated molecular patterns
EVs: extracellular vesicles
HTG: hypertriglyceridemia
lncRNA: long non-coding RNA
miRNA: microRNA
MSCs: mesenchymal stem cells
SAP: severe acute pancreatitis

## References

[1] Mederos MA, Reber HA, Girgis MD (2021). Acute Pancreatitis: A Review. Jama.

[2] Granger J, Remick D (2005). Acute pancreatitis: models, markers, and mediators. Shock.

[3] Tenner S, Vege SS, Sheth SG, Sauer B, Yang A, Conwell DL (2024). American College of Gastroenterology Guidelines: Management of Acute Pancreatitis. Am J Gastroenterol.

[4] Lugea A, Waldron RT, Mareninova OA, Shalbueva N, Deng N, Su HY (2017). Human Pancreatic Acinar Cells: Proteomic Characterization, Physiologic Responses, and Organellar Disorders in ex Vivo Pancreatitis. Am J Pathol.

[5] Gukovskaya AS, Pandol SJ, Gukovsky I (2016). New insights into the pathways initiating and driving pancreatitis. Curr Opin Gastroenterol.

[6] Petersen OH, Gerasimenko OV, Tepikin AV, Gerasimenko JV (2011). Aberrant Ca(2+) signalling through acidic calcium stores in pancreatic acinar cells. Cell Calcium.

[7] Romac JM, Shahid RA, Swain SM, Vigna SR, Liddle RA (2018). Piezo1 is a mechanically activated ion channel and mediates pressure induced pancreatitis. Nat Commun.

[8] Wen L, Voronina S, Javed MA, Awais M, Szatmary P, Latawiec D (2015). Inhibitors of ORAI1 Prevent Cytosolic Calcium-Associated Injury of Human Pancreatic Acinar Cells and Acute Pancreatitis in 3 Mouse Models. Gastroenterology.

[9] Dawra R, Sah RP, Dudeja V, Rishi L, Talukdar R, Garg P, Saluja AK (2011). Intra-acinar trypsinogen activation mediates early stages of pancreatic injury but not inflammation in mice with acute pancreatitis. Gastroenterology.

[10] Halangk W, Lerch MM, Brandt-Nedelev B, Roth W, Ruthenbuerger M, Reinheckel T (2000). Role of cathepsin B in intracellular trypsinogen activation and the onset of acute pancreatitis. J Clin Invest.

[11] Habtezion A (2015). Inflammation in acute and chronic pancreatitis. Curr Opin Gastroenterol.

[12] Hu F, Lou N, Jiao J, Guo F, Xiang H, Shang D (2020). Macrophages in pancreatitis: Mechanisms and therapeutic potential. Biomed Pharmacother.

[13] Shrivastava P, Bhatia M (2010). Essential role of monocytes and macrophages in the progression of acute pancreatitis. World J Gastroenterol.

[14] Cosentino C, Regazzi R (2021). Crosstalk between Macrophages and Pancreatic β-Cells in Islet Development, Homeostasis and Disease. Int J Mol Sci.

[15] Baer JM, Zuo C, Kang LI, de la Lastra AA, Borcherding NC, Knolhoff BL (2023). Fibrosis induced by resident macrophages has divergent roles in pancreas inflammatory injury and PDAC. Nat Immunol.

[16] Ying W, Lee YS, Dong Y, Seidman JS, Yang M, Isaac R (2019). Expansion of Islet-Resident Macrophages Leads to Inflammation Affecting β Cell Proliferation and Function in Obesity. Cell Metab.

[17] Lazarov T, Juarez-Carreño S, Cox N, Geissmann F (2023). Physiology and diseases of tissue-resident macrophages. Nature.

[18] Hoque R, Sohail M, Malik A, Sarwar S, Luo Y, Shah A (2011). TLR9 and the NLRP3 inflammasome link acinar cell death with inflammation in acute pancreatitis. Gastroenterology.

[19] Peng Y, Li Y, Yang Y, Shi T, Liu R, Luan Y, Yin C (2023). The Role and Potential Regulatory Mechanism of STING Modulated Macrophage Apoptosis and Differentiation in Severe Acute Pancreatitis-Associated Lung Injury. J Interferon Cytokine Res.

[20] Zhao Q, Wei Y, Pandol SJ, Li L, Habtezion A (2018). STING Signaling Promotes Inflammation in Experimental Acute Pancreatitis. Gastroenterology.

[21] Peng C, Tu G, Wang J, Wang Y, Wu P, Yu L (2023). MLKL signaling regulates macrophage polarization in acute pancreatitis through CXCL10. Cell Death Dis.

[22] Liu J, Niu Z, Zhang R, Peng Z, Wang L, Liu Z (2021). MALAT1 shuttled by extracellular vesicles promotes M1 polarization of macrophages to induce acute pancreatitis via miR-181a-5p/HMGB1 axis. J Cell Mol Med.

[23] Tang DS, Cao F, Yan CS, Cui JT, Guo XY, Cheng L (2022). Acinar Cell-Derived Extracellular Vesicle MiRNA-183-5p Aggravates Acute Pancreatitis by Promoting M1 Macrophage Polarization Through Downregulation of FoxO1. Front Immunol.

[24] Zheng Z, Cao F, Ding YX, Lu JD, Fu YQ, Liu L (2023). Acinous cell AR42J-derived exosome miR125b-5p promotes acute pancreatitis exacerbation by inhibiting M2 macrophage polarization via PI3K/AKT signaling pathway. World J Gastrointest Surg.

[25] Su XJ, Chen Y, Zhang QC, Peng XB, Liu YP, Wang L, Du YQ (2024). Exosomes Derived from Caerulein-Stimulated Pancreatic Acinar Cells Mediate Peritoneal Macrophage M1 Polarization and Pyroptosis via an miR-24-3p/MARCH3/NLRP3 Axis in Acute Pancreatitis. Pancreas.

[26] Sendler M, Weiss FU, Golchert J, Homuth G, van den Brandt C, Mahajan UM (2018). Cathepsin B-Mediated Activation of Trypsinogen in Endocytosing Macrophages Increases Severity of Pancreatitis in Mice. Gastroenterology.

[27] Xu T, Sheng L, Guo X, Ding Z (2022). Free Fatty Acid Increases the Expression of NLRP3-Caspase1 in Adipose Tissue Macrophages in Obese Severe Acute Pancreatitis. Dig Dis Sci.

[28] Bonjoch L, Gea-Sorlí S, Closa D (2015). Lipids generated during acute pancreatitis increase inflammatory status of macrophages by interfering with their M2 polarization. Pancreatology.

[29] Hu N, Zhang X, Zhang X, Guan Y, He R, Xue E (2022). Inhibition of Notch activity suppresses hyperglycemia-augmented polarization of macrophages to the M1 phenotype and alleviates acute pancreatitis. Clin Sci (Lond).

[30] Pan Y, Li Y, Gao L, Tong Z, Ye B, Liu S (2017). Development of a novel model of hypertriglyceridemic acute pancreatitis in mice. Sci Rep.

[31] Pan Y, Cao S, Tang J, Arroyo JP, Terker AS, Wang Y (2022). Cyclooxygenase-2 in adipose tissue macrophages limits adipose tissue dysfunction in obese mice. J Clin Invest.

[32] Cho KW, Zamarron BF, Muir LA, Singer K, Porsche CE, DelProposto JB (2016). Adipose Tissue Dendritic Cells Are Independent Contributors to Obesity-Induced Inflammation and Insulin Resistance. J Immunol.

[33] Haase J, Weyer U, Immig K, Klöting N, Blüher M, Eilers J (2014). Local proliferation of macrophages in adipose tissue during obesity-induced inflammation. Diabetologia.

[34] Jiang D, Wang J, Guo S (2022). Correlations of Triglyceride with Type, Severity and Clinical Prognosis of Acute Pancreatitis. Clin Lab.

[35] Farquhar R, Matthews S, Baxter N, Rayers G, Ratnayake CBB, Robertson FP (2023). Sarcopenia and Sarcopenic Obesity on Body Composition Analysis is a Significant Predictor of Mortality in Severe Acute Pancreatitis: A Longitudinal Observational Study. World J Surg.

[36] Han H, Zhang L, Fu Q, Zhang B, Chen J (2023). Plasma Exosomes Aggravate Acute Pancreatitis by Promoting M1 Polarization of Adipose Tissue Macrophages in Obesity-Related Severe Acute Pancreatitis. Dig Dis Sci.

[37] Xu L, Yang F, Lin R, Han C, Liu J, Ding Z (2014). Induction of m2 polarization in primary culture liver macrophages from rats with acute pancreatitis. PLoS One.

[38] Liu RH, Wen Y, Sun HY, Liu CY, Zhang YF, Yang Y (2018). Abdominal paracentesis drainage ameliorates severe acute pancreatitis in rats by regulating the polarization of peritoneal macrophages. World J Gastroenterol.

[39] Yuan X, Luo C, Wu J, Li W, Guo X, Li S (2021). Abdominal paracentesis drainage attenuates intestinal mucosal barrier damage through macrophage polarization in severe acute pancreatitis. Exp Biol Med (Maywood).

[40] Sun K, He SB, Qu JG, Dang SC, Chen JX, Gong AH (2016). IRF5 regulates lung macrophages M2 polarization during severe acute pancreatitis in vitro. World J Gastroenterol.

[41] Wen E, Xin G, Li S, Dong Y, Zhu Y, Wan C (2022). Tuftsin ameliorates splenic inflammatory injury by promoting neuropilin-1 in severe acute pancreatitis. Biochem Pharmacol.

[42] Sheng LP, Han CQ, Ling X, Guo XW, Lin R, Ding Z (2023). Proanthocyanidins suppress NLRP3 inflammasome and M1 macrophage polarization to alleviate severe acute pancreatitis in mice. J Biochem Mol Toxicol.

[43] Wu J, Zhang L, Shi J, He R, Yang W, Habtezion A (2020). Macrophage phenotypic switch orchestrates the inflammation and repair/regeneration following acute pancreatitis injury. EBioMedicine.

[44] Lu Y, Lu G, Gao L, Zhu Q, Xue J, Zhang J (2021). The Proresolving Lipid Mediator Maresin1 Alleviates Experimental Pancreatitis via Switching Macrophage Polarization. Mediators Inflamm.

[45] Yuan C, Xu X, Wang N, Zhu Q, Zhang J, Gong W (2022). Paeonol protects against acute pancreatitis by inhibiting M1 macrophage polarization via the NLRP3 inflammasomes pathway. Biochem Biophys Res Commun.

[46] Wang J, Tian J, Wang L, Yang ZW, Xu P (2023). Mesenchymal stem cells regulate M1 polarization of peritoneal macrophages through the CARD9-NF-κB signaling pathway in severe acute pancreatitis. J Hepatobiliary Pancreat Sci.

[47] Peng K, Biao C, Zhao YY, Jun LC, Wei W, YLNYZ ABLZ, Song L (2023). Long non-coding RNA MM2P suppresses M1-polarized macrophages-mediated excessive inflammation to prevent sodium taurocholate-induced acute pancreatitis by blocking SHP2-mediated STAT3 dephosphorylation. Clin Exp Med.

[48] Ren W, Zhao L, Sun Y, Wang X, Shi X (2023). HMGB1 and Toll-like receptors: potential therapeutic targets in autoimmune diseases. Mol Med.

[49] Ablasser A, Chen ZJ (2019). cGAS in action: Expanding roles in immunity and inflammation. Science.

[50] Murai S, Yamaguchi Y, Shirasaki Y, Yamagishi M, Shindo R, Hildebrand JM (2018). A FRET biosensor for necroptosis uncovers two different modes of the release of DAMPs. Nat Commun.

[51] Danielski LG, Giustina AD, Bonfante S, Barichello T, Petronilho F (2020). The NLRP3 Inflammasome and Its Role in Sepsis Development. Inflammation.

[52] Pan Y, You Y, Sun L, Sui Q, Liu L, Yuan H (2021). The STING antagonist H-151 ameliorates psoriasis via suppression of STING/NF-κB-mediated inflammation. Br J Pharmacol.

[53] Zhang Y, Jing Y, Liao H, Ding K, Zhang L, Liao S (2026). STING deficiency alleviates ischemia-reperfusion injury via JAK2/STAT3-mediated macrophage polarization and autophagy. Int Immunopharmacol.

[54] Liu Z, Lei Y, Zuo J, Zhang R, Du H, Hu H (2025). Activated Notch1 promotes macrophage polarization and exacerbates sepsis-induced acute lung injury via β-catenin/NF-κB signaling. Biochem Pharmacol.

[55] Duan F, Wang X, Wang H, Wang Y, Zhang Y, Chen J (2022). GDF11 ameliorates severe acute pancreatitis through modulating macrophage M1 and M2 polarization by targeting the TGFβR1/SMAD-2 pathway. Int Immunopharmacol.

[56] Huang Q, Cheng X, Luo C, Yang S, Li S, Wang B (2021). Placental chorionic plate-derived mesenchymal stem cells ameliorate severe acute pancreatitis by regulating macrophage polarization via secreting TSG-6. Stem Cell Res Ther.

[57] Zhou L, Yu J, Wang S, Ma Y, Liu X, Zhang X (2023). Tectoridin alleviates caerulein-induced severe acute pancreatitis by targeting ERK2 to promote macrophage M2 polarization. Arch Biochem Biophys.

[58] Zhang D, Li L, Li J, Wei Y, Tang J, Man X, Liu F (2022). Colchicine improves severe acute pancreatitis-induced acute lung injury by suppressing inflammation, apoptosis and oxidative stress in rats. Biomed Pharmacother.

[59] Cao Y, Adhikari S, Clément MV, Wallig M, Bhatia M (2007). Induction of apoptosis by crambene protects mice against acute pancreatitis via anti-inflammatory pathways. Am J Pathol.

[60] Zhao X, Jin B, Yang B, Yan W, Wu X, Jiang C, Cheng S (2017). Gadolinium chloride ameliorates acute lung injury associated with severe acute pancreatitis in rats by regulating CYLD/NF-κB signaling. Biochem Biophys Res Commun.

[61] Wang Y, Cao C, Zhu Y, Fan H, Liu Q, Liu Y (2022). TREM2/β-catenin attenuates NLRP3 inflammasome-mediated macrophage pyroptosis to promote bacterial clearance of pyogenic bacteria. Cell Death Dis.

[62] Wu XB, Sun HY, Luo ZL, Cheng L, Duan XM, Ren JD (2020). Plasma-derived exosomes contribute to pancreatitis-associated lung injury by triggering NLRP3-dependent pyroptosis in alveolar macrophages. Biochim Biophys Acta Mol Basis Dis.

[63] Peng Y, Yang Y, Li Y, Shi T, Xu N, Liu R (2024). Mitochondrial (mt)DNA-cyclic GMP-AMP synthase (cGAS)-stimulator of interferon genes (STING) signaling promotes pyroptosis of macrophages via interferon regulatory factor (IRF)7/IRF3 activation to aggravate lung injury during severe acute pancreatitis. Cell Mol Biol Lett.

[64] Wu X, Yao J, Hu Q, Kang H, Miao Y, Zhu L (2022). Emodin Ameliorates Acute Pancreatitis-Associated Lung Injury Through Inhibiting the Alveolar Macrophages Pyroptosis. Front Pharmacol.

[65] Li H, Lin Y, Zhang L, Zhao J, Li P (2022). Ferroptosis and its emerging roles in acute pancreatitis. Chin Med J (Engl).

[66] Liu K, Liu J, Zou B, Li C, Zeh HJ, Kang R (2022). Trypsin-Mediated Sensitization to Ferroptosis Increases the Severity of Pancreatitis in Mice. Cell Mol Gastroenterol Hepatol.

[67] Fortunato F, Bürgers H, Bergmann F, Rieger P, Büchler MW, Kroemer G, Werner J (2009). Impaired autolysosome formation correlates with Lamp-2 depletion: role of apoptosis, autophagy, and necrosis in pancreatitis. Gastroenterology.

[68] Mareninova OA, Sendler M, Malla SR, Yakubov I, French SW, Tokhtaeva E (2015). Lysosome associated membrane proteins maintain pancreatic acinar cell homeostasis: LAMP-2 deficient mice develop pancreatitis. Cell Mol Gastroenterol Hepatol.

[69] Fan HN, Chen W, Fan LN, Wu JT, Zhu JS, Zhang J (2019). Macrophages-derived p38α promotes the experimental severe acute pancreatitis by regulating inflammation and autophagy. Int Immunopharmacol.

[70] Sheng LP, Shang GC, Han CQ, Ling X, Guo XW, Lin R, Ding Z (2026). Impaired Autophagic Flux in Adipose Tissue Aggravates Pancreatic Injury in Obesity-Related Severe Acute Pancreatitis. Immun Inflamm Dis.

[71] Ling X, Zhang Z, Lin L, Guo X, Ding Z (2025). Dysfunction of Autophagy in Adipose Tissue Macrophages Regulated via FoxO1 in Obesity-Related Severe Acute Pancreatitis. Int J Mol Sci.

